# Dysregulation of lipid metabolism, energy production, and oxidative stress in myalgic encephalomyelitis/chronic fatigue syndrome, Gulf War Syndrome and fibromyalgia

**DOI:** 10.3389/fnins.2025.1498981

**Published:** 2025-03-10

**Authors:** Leah Davis, Maisy Higgs, Ailsa Snaith, Tiffany A. Lodge, James Strong, Jose A. Espejo-Oltra, Sławomir Kujawski, Paweł Zalewski, Etheresia Pretorius, Michael Hoerger, Karl J. Morten

**Affiliations:** ^1^The Nuffield Department of Women's and Reproductive Health, The Women Centre, The John Radcliffe Hospital, The University of Oxford, Oxford, United Kingdom; ^2^Veterans and Families Institute for Military Social Research, Anglia Ruskin University, Chelmsford, United Kingdom; ^3^Department of Pathology, Catholic University of Valencia Saint Vincent Martyr, Valencia, Spain; ^4^Department of Exercise Physiology and Functional Anatomy, Collegium Medicum in Bydgoszcz, Bydgoszcz, Poland, Nicolaus Copernicus University in Torun, Torun, Poland; ^5^Department of Experimental and Clinical Physiology, Warsaw Medical University, Warszawa, Poland; ^6^Department of Physiological Sciences, Faculty of Science, Stellenbosch University, Stellenbosch, South Africa; ^7^Department of Biochemistry and Systems Biology, Institute of Systems, Molecular and Integrative Biology, Faculty of Health and Life Sciences, University of Liverpool, Liverpool, United Kingdom; ^8^Departments of Psychology, Psychiatry, and Medicine, Tulane Cancer Center, Tulane University, New Orleans, LA, United States

**Keywords:** oxidative stress, ME/CFS, Gulf War Syndrome, fibromyalgia, energy metabolism, lipid dysregulation

## Abstract

Myalgic encephalomyelitis/chronic fatigue syndrome (ME/CFS), Gulf War Syndrome (GWS), and Fibromyalgia (FM) are complex, chronic illnesses with overlapping clinical features. Symptoms that are reported across these conditions include post-exertional malaise (PEM), fatigue, and pain, yet the etiology of these illnesses remains largely unknown. Diagnosis is challenging in patients with these conditions as definitive biomarkers are lacking; patients are required to meet clinical criteria and often undergo lengthy testing to exclude other conditions, a process that is often prolonged, costly, and burdensome for patients. The identification of reliable validated biomarkers could facilitate earlier and more accurate diagnosis and drive the development of targeted pharmacological therapies that might address the underlying pathophysiology of these diseases. Major driving forces for biomarker identification are the advancing fields of metabolomics and proteomics that allow for comprehensive characterization of metabolites and proteins in biological specimens. Recent technological developments in these areas enable high-throughput analysis of thousands of metabolites and proteins from a variety of biological samples and model systems, that provides a powerful approach to unraveling the metabolic phenotypes associated with these complex diseases. Emerging evidence suggests that ME/CFS, GWS, and FM are all characterized by disturbances in metabolic pathways, particularly those related to energy production, lipid metabolism, and oxidative stress. Altered levels of key metabolites in these pathways have been reported in studies highlighting potential common biochemical abnormalities. The precise mechanisms driving altered metabolic pathways in ME/CFS, GWS, and FM remain to be elucidated; however, the elevated oxidative stress observed across these illnesses may contribute to symptoms and offer a potential target for therapeutic intervention. Investigating the mechanisms, and their role in the disease process, could provide insights into disease pathogenesis and reveal novel treatment targets. As such, comprehensive metabolomic and proteomic analyses are crucial for advancing the understanding of these conditions in-order to identify both common, and unique, metabolic alterations that could serve as diagnostic markers or therapeutic targets.

## Introduction

**Figure 1:**
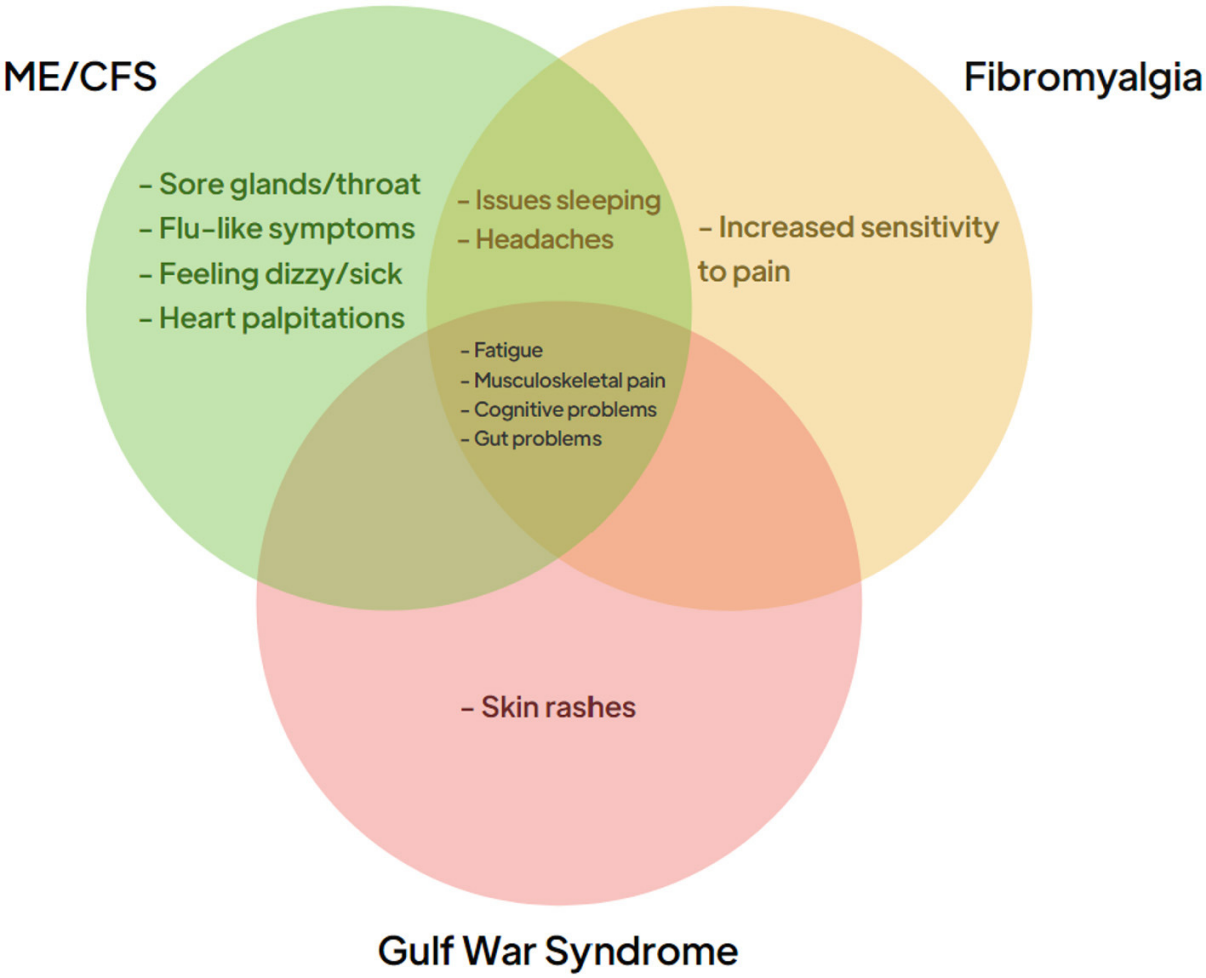
Clinical symptoms of chronic conditions. The symptoms associated with the three chronic conditions ME/CFS (green), Fibromyalgia (orange), and Gulf War Syndrome (red). The central panel identifies the clinical symptoms that often present themselves in patients suffering from all three conditions.

**Figure 2:**
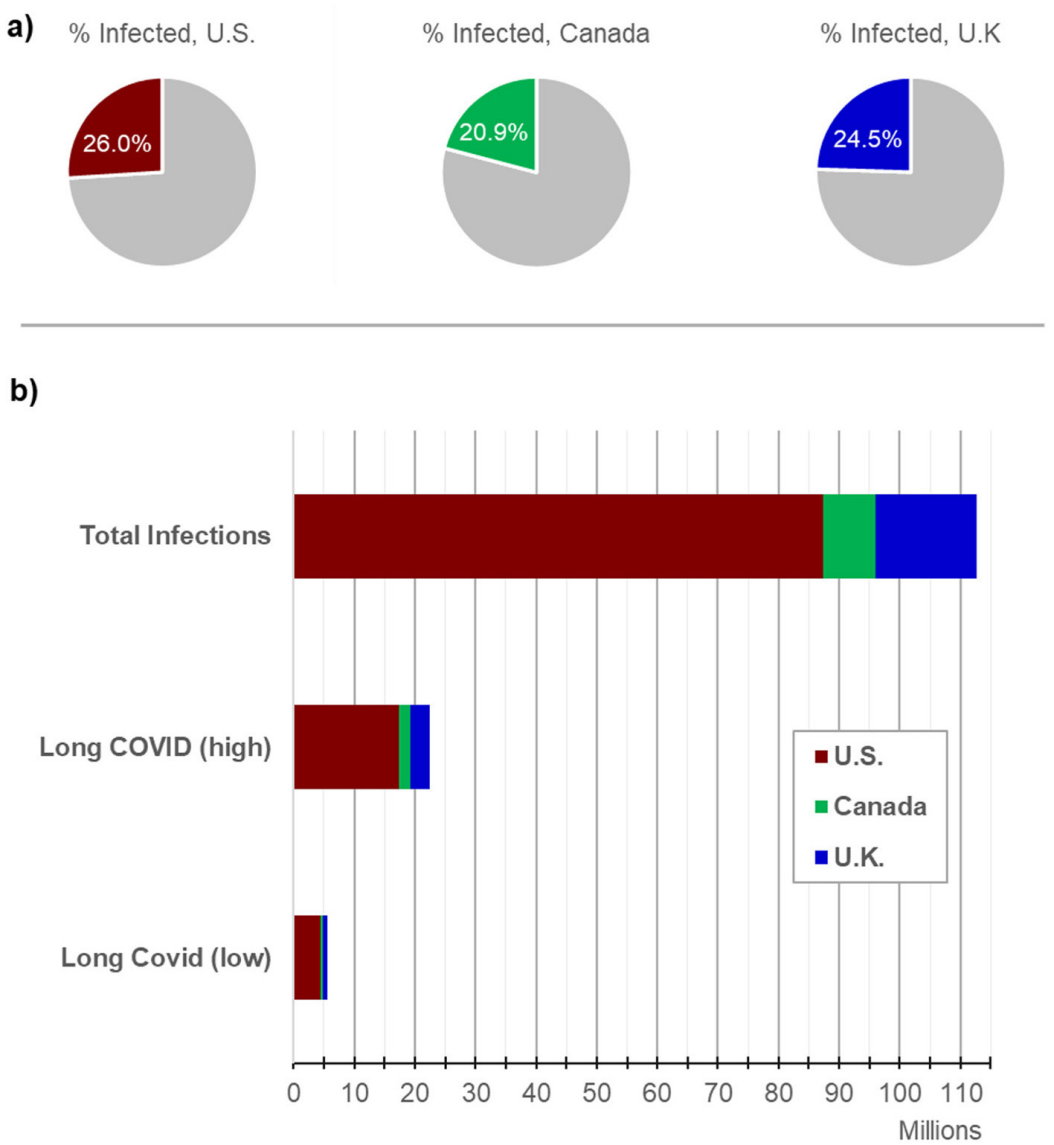
COVID burden during the peak 2 months of the winter 2023–24 wave. COVID data were most readily available internationally during the winter 2023–24 wave. Across the U.S., Canada, and U.K., a similar proportion of residents were estimated to get infected with COVID [**(A)**, 20.9–26.0%]. Estimated total infections surpassed 112 million in these nations during the peak 2 months of the wave **(B)**. With an estimated 5–20% of infections resulting in Long COVID (low and high estimates), 5.6–22.5 million of these residents would develop a new Long COVID condition from that wave alone.

Approximately one in three adults worldwide is estimated to suffer from one or more chronic illnesses, imposing a substantial burden on both the patients and healthcare systems globally. These complex chronic conditions are associated with a marked reduction in the quality of life for affected individuals and lead to increased healthcare costs due to the need for ongoing management and intervention (Hajat and Stein, 2018). Chronic illnesses are typically defined as long-term health conditions that are persistent and require continuous management, often through a combination of pharmacological and non-pharmacological therapies. Fatigue is a common symptom in many chronic illnesses and can be debilitating; some patients become so severely affected to the point of being bedbound (Goërtz et al., 2021). Conditions such as Myalgic encephalomyelitis/chronic fatigue syndrome (ME/CFS), Fibromyalgia (FM), and Gulf War Syndrome (GWS) are examples of these chronic illnesses that are triggered by different factors but share overlapping clinical symptoms, in particular profound fatigue (see Figure 1). The diagnosis of ME/CFS and FM is inherently complex, and often one of exclusion. Many individuals with ME/CFS and FM remain undiagnosed, or misdiagnosed, due to overlapping symptoms with other conditions and insufficiently specific diagnostic criteria. Efforts to improve study cohorts have included the adoption of standardized criteria such as the Canadian Consensus Criteria (CCC) and the CDC-1994 criteria (Carruthers et al., 2003; Fukuda et al., 1994); however, even within these frameworks, heterogeneity within recruited populations remains a significant limitation. Misdiagnoses, and the variability in symptom severity within ME/CFS cohorts, likely influences study outcomes aimed at identification of disease-associated biomarkers (Xu et al., 2023). Malato et al. (2023) estimated that over 1,000 participants per group would be required in ME/CFS studies to account for misclassification, a scale beyond current studies (Malato et al., 2023). Similarly, FM studies face challenges in patient recruitment due to diagnostic inaccuracies. While the International Classification of Diseases most recent iteration (ICD-11) categorizes FM under MG30.01—“chronic widespread pain”, patients are often diagnosed years after symptom onset or incorrectly assigned an FM diagnosis, and this likely has an impact on research outcomes. GWS affects 25–30% of military veterans that served in the 1990–91 Gulf war; the condition, with its links to a specific conflict, offers a more defined trigger, likely due to exposure to toxic agents such as organophosphates, nerve agents, and pyridostigmine bromide. In contrast with the circumscribed triggers of GWS, ME/CFS is associated with a number of potential infectious triggers, while Epstein-Barr virus (EBV) has been hypothesized as a contributor to FM. Long COVID, studied widely in recent years, also shares common features with these conditions, and this further complicates the landscape, as studies indicate that 20–31% of Long COVID patients also meet FM diagnostic criteria 6 months post-infection (Fernández-de-Las-Peñas et al., 2022; Ursini et al., 2021). Furthermore, there may also be a contribution made by reactivation of latent viruses. In particular, EBV reactivation is observed in ME/CFS (Koelle et al., 2002; Ruiz-Pablos et al., 2021), FM (Duffy et al., 2022), and Long COVID (Shafiee et al., 2023), suggesting a possible shared pathological pathway. The RECOVER study, published in 2025, highlighted that ME/CFS diagnoses have increased significantly since the COVID pandemic, with many long COVID patients now meeting ME/CFS criteria (Vernon et al., 2025). Wastewater surveillance data in the U.S. show 10 COVID waves thus far, with an average of one infection per individual every 13.7 months (Hoerger, 2025). The Winter 2023–24 wave was monitored by multiple international surveillance models, which reported similar infection rates during the peak 2 months in the U.S., Canada, and the U.K. (Hoerger et al., 2025). Based on standard estimates that 5–20% of infections lead to new Long COVID conditions, this wave alone (see Figure 2) may have resulted in ~5.6 to 22.5 million new cases of long COVID (Hoerger, 2025; Geng et al., 2025; Xie et al., 2024).

To be diagnosed with GWS, the Centers for Disease Control and Prevention (CDC) criteria for Chronic Multisymptomatic Illness (CMI) require symptoms from at least two of the three symptom clusters: general fatigue, mood and cognitive abnormalities, and myalgia/athralgia pain (Fukuda et al., 1998). The chronic symptoms are consistent with Chronic Organophosphate-Induced Neuropsychiatric Disorder (COPIND) (Namba et al., 1971; Richardson et al., 2020). COPIND is associated with long term symptoms resulting from prolonged or repeated exposure organophosphates which are chemicals found in pesticides, nerve agents and some industrial products. The routes of potential exposure to these chemicals are clearly not solely the preserve of the military environment, thus associated symptoms such as fatigue are prevalent across parts of society. For an excellent review of diagnostic issues in fatigue-associated conditions and brain effects, see Baraniuk (2022). GWS is specifically linked with exposure to neurotoxic agents, including organophosphates, carbamates, sarin/cyclosarin nerve agents, and pyridostigmine bromide, used as prophylactic measures against chemical warfare, whereas ME/CFS has been associated with viral infection triggers. Similarly, a potential trigger for FM has been suggested to be the Epstein-Barr virus (Cohen et al., 2022). FM is associated with chronic musculoskeletal pain with patients also suffering from fatigue, sleep, and cognitive disturbances. Like ME/CFS, FM more commonly affects women. ME/CFS, FM, and GWS were selected for this review due to (i) the common overlap of ME/CFS and FM where similar biological dysfunction might be expected and (ii) GWS, with its identifiable and specific trigger, likely to offer a different biological perspective. A major challenge in these conditions, and other chronic diseases with unknown causes, is identifying the biological changes that are driving symptoms, thus enabling the identification of new treatment targets. It is conceivable that once a treatment is identified for one of these conditions, that may share biological mechanisms, it could have potential relevance across a wider range of conditions. Although not covered in this review Long COVID, triggered by SARS-CoV-2 infection, which affects many millions of people globally, is another condition likely to have many common features with ME/CFS (Komaroff and Lipkin, 2023), FM, and GWS. For example, 20%−31% of patients with Long COVID met the criteria for fibromyalgia more than 6 months after their COVID-19 infection (Fernández-de-Las-Peñas et al., 2022; Ursini et al., 2021). Reactivation of latent viruses, particularly the Epstein-Barr virus, has been observed in conditions such as ME/CFS (Koelle et al., 2002; Ruiz-Pablos et al., 2021), FM (Duffy et al., 2022) and Long COVID (Shafiee et al., 2023; Peluso et al., 2023). Nevertheless, an overlap in biological mechanisms underlying those conditions is not fully understood (Goldenberg, 2024).

Despite differing aetiological factors, the clinical presentation of ME/CFS, FM, and GWS demonstrates many overlapping characteristics suggesting the possibility of common biological pathways. Fatigue is a central feature of all these disorders, often exacerbated by exertion and non-restorative sleep. Fatigue can be categorized as chronic when it persists for more than 6 months and is unrelieved by rest, often without a clear underlying medical cause. Secondary fatigue tends to be linked to identifiable medical conditions and typically lasts <6 months, whereas physiological fatigue results from imbalances in sleep, nutrition, and physical activity and is relieved by rest. Fatigue is a common symptom in both ME/CFS and Fibromyalgia, with post-exertional malaise (PEM) being a hallmark of ME/CFS. PEM is a distinct clinical presentation characterized by symptom exacerbation following physical or cognitive exertion. The severity of PEM varies widely, with recovery times ranging from hours to weeks, and it has been shown that in CFS patients who have symptoms of PEM, a physical exercise programme may lead to the worsening of their symptoms (Vink and Vink-Niese, 2020). Jason et al. (2021) have developed a validated questionnaire for subjective PEM assessment (Jason et al., 2021) which has been translated into multiple languages. In addition, research has demonstrated that inflammatory biomarker differences have been observed based on PEM severity, indicating that distinct physiological mechanisms may underlie the presentation (White et al., 2010). Similar PEM-like symptoms, including exertional exhaustion and post-exertion symptom exacerbation, are also reported in GWS patients (Baraniuk, 2022) and FM (McManimen and Jason, 2017), with co-morbid FM and ME/CFS resulting in increased illness severity and greater symptoms burden (McManimen and Jason, 2017). The worsening of symptoms, or the emergence of new symptoms following exertion, provides a valuable opportunity to investigate the underlying metabolic dysfunction associated with these conditions. Current clinical diagnostic tools are often inadequate for identifying the underlying mechanisms driving these symptoms, and this is a reflection of the broader challenge of understanding the complex and multifactorial nature of these disorders (Rosenthal et al., 2008).

**Figure 3:**
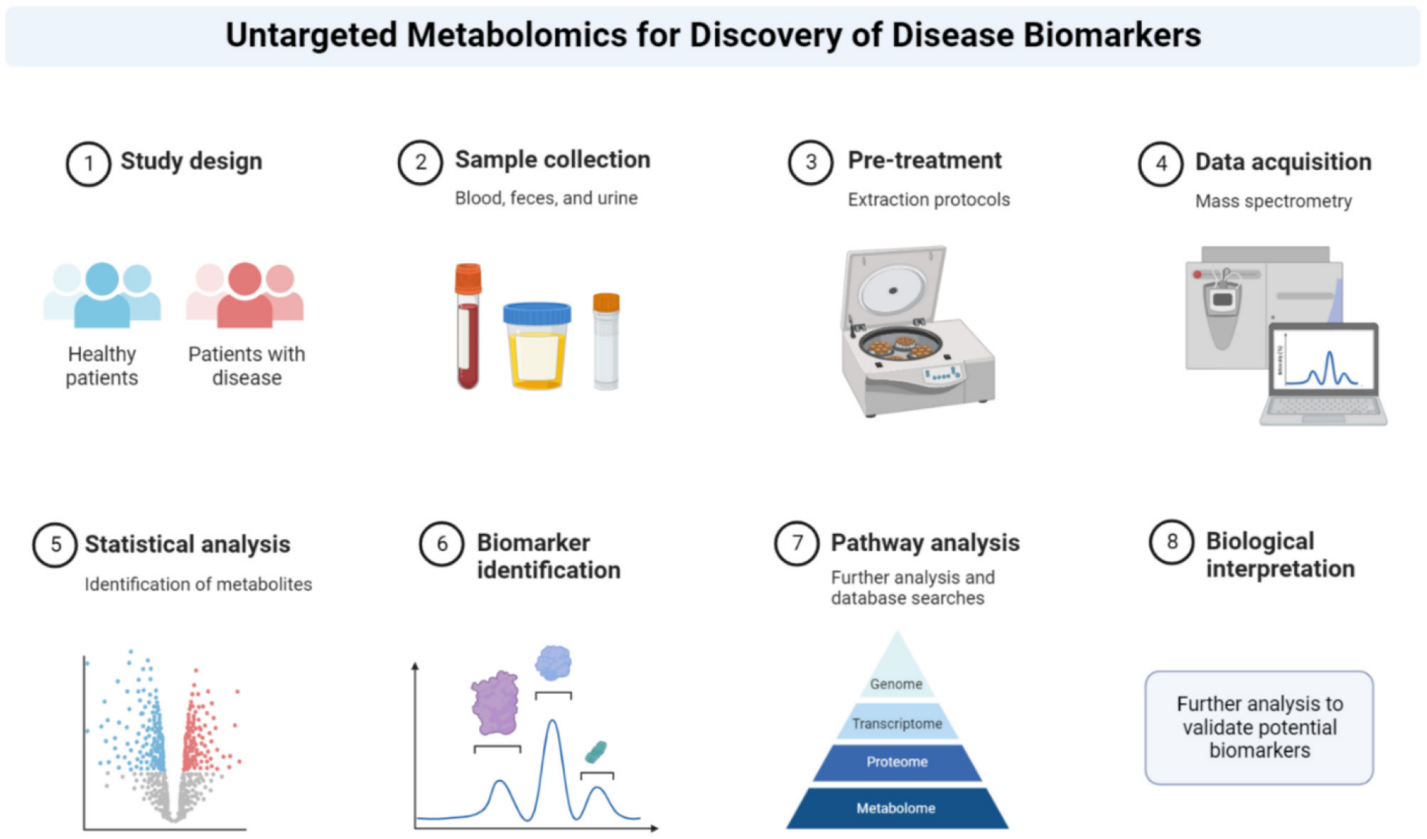
Metabolomics workflow. Summary of metabolomics protocol. The method describes how the patient sample is retrieved, analyzed and interpreted. Reprinted from “Untargeted Metabolomics for Discovery of Disease Biomarkers”, by BioRender.com (2022). Retrieved from https://app.biorender.com/biorender-templates.

Metabolomics is the comprehensive study of metabolites, the small molecules that are intermediates and end products of cellular metabolism. Metabolite levels are influenced by both genetic and environmental factors, providing a snapshot of the biochemical activity within cells and tissues. Large-scale metabolic profiling can be conducted (Figure 3) using a variety of biological samples, including specific cells, biological fluids, and tissues, generating detailed metabolic reports. As of 2022, the Human Metabolome Database (HMDB Version 5.0) contained 217,920 annotated metabolites, with a further 1,581,537 unannotated derivatized metabolite entries from gas chromatography-mass spectrometry (GC-MS) data (Wishart et al., 2022). Advances in metabolite processing methods and metabolite identification tools, such as Bio-Transformer, are expected to further expand the number of annotated metabolites, enhancing the comprehensive coverage enabled by of metabolic databases (Djoumbou-Feunang et al., 2019; Walsby-Tickle et al., 2020). A range of analytical techniques, including Nuclear Magnetic Resonance (NMR) spectroscopy, High-performance liquid chromatography (HPLC), and combinations of mass spectrometry (MS) with liquid or gas chromatography (LC, GC) can be employed to capture the metabolic profile of samples; however, these methods still only provide partial coverage of the metabolome (Wishart, 2011). Variation in analytical techniques, and the increasing number of identified metabolites, complicate comparisons between studies, particularly those conducted several years apart. For example, Metabolon, a major commercial provider of metabolomics services widely used in the United States, employs a general metabolite panel and a more specific lipid-focused panel, which can lead to inconsistencies when comparing results across different platforms or timeframes (Germain et al., 2020). Despite its potential, the full capabilities of metabolomics in biomarker discovery and understanding disease pathogenesis have yet to be realized. Compared with other “-omics” approaches, such as transcriptomics and proteomics, the application of metabolomics in studying chronic conditions like ME/CFS, is still emerging. Nevertheless, metabolomics offers a distinct advantage due to its direct representation of the biochemical phenotypes, which is particularly valuable in chronic conditions without established diagnostic tests. By comparing metabolite profiles across different patient cohorts, common dysregulated pathways can be identified, potentially revealing the underlying clinical pathology (Fiehn, 2002; Arjmand et al., 2022; Sun C. et al., 2020; Sun Y. et al., 2020; Voss et al., 2021). This approach is especially relevant in the context of chronic illnesses where the identification of specific biomarkers could lead to better diagnostic and therapeutic strategies. For example, ongoing research has explored the use of single-cell transcriptomics to establish immune system baselines in ME/CFS patients to compare ME/CFS patients and to detect deviations following cardiopulmonary exercise testing (Vu et al., 2024). Similarly, metabolomics has shown promise in expanding our understanding of other prevalent conditions, such as cardiovascular diseases (Shah et al., 2012), obesity (Chen et al., 2015), and type 2 diabetes (Zheng and Hu, 2015).

The integration of metabolomics into chronic disease research holds the potential to uncover novel biomarkers as therapeutic targets, thereby advancing personalized medicine and improving patient outcomes (Karczewski and Snyder, 2018). This review aims to compare the plasma and serum metabolomics profiles of patients with ME/CFS, FM, and GWS. While ME/CFS and FM often co-occur, and GWS has distinct initial triggers, these conditions share numerous overlapping symptoms. Later in the review, we look for supporting evidence for metabolic pathway dysfunction, identified by serum and plasma metabolomics, by examining proteomics analysis of biological material and whole patient imaging studies. Our aim is not to produce a list of defective pathways but to identify common patterns/pathways which may underpin a common etiology in these three conditions. Although identifying the initial triggers for ME/CFS and FM is crucial, a more pressing concern for patients is understanding the biological pathways that currently underpin their symptoms. By identifying common dysregulated pathways across these conditions, we can gain insights into the complex pathophysiology underlying symptoms such as fatigue, PEM and postural orthostatic tachycardia syndrome (POTS). POTS is a condition characterized by a disproportionate increase in heart rate on standing, and is often accompanied by symptoms life dizziness, light-headedness, and fatigue. PEM and orthostatic intolerance, including POTS, are recognized as cardinal symptoms in ME/CFS patients (Committee on the Diagnostic Criteria for Myalgic Encephalomyelitis/Chronic Fatigue Syndrome; Board on the Health of Select Populations; Institute of Medicine, 2015). In a study of 3,933 respondents diagnosed with POTS, 20% also had FM and 21% had ME/CFS, highlighting the overlap between these conditions (Vernino et al., 2021). PEM, which is characterized by an exacerbation of symptoms following physical or cognitive exertion, is also observed in FM and may be related to increased pain severity post-exertion (Barhorst et al., 2022). Furthermore, transient POTS has been reported in approximately one-third of GWS patients, with a potential association with PEM (Rayhan et al., 2013). These findings suggest that both POTS and PEM are common features in ME/CFS, Fibromyalgia, and GWS.

## Metabolite similarities and proposed functional consequences

As outlined earlier, the chronic conditions described in this review share a range of similar symptoms as summarized in Figure 1. Analyzing the metabolic profiles of these patient groups may reveal commonly dysregulated pathways that contribute to these shared symptoms. Future longitudinal studies that correlate symptoms fluctuations with alterations in these pathways could also help identify new therapeutic targets. If a small number of pathways are consistently dysregulated across these conditions, targeting these pathways could potentially provide symptom relief for a broad spectrum of patients with different diagnoses. In the subsequent sections, we will compare the metabolomic profiles of GWS and FM to those of ME/CFS, aiming to identify any common pathways that are dysregulated across these conditions.

### ME/CFS and Gulf War Syndrome

**Figure 4:**
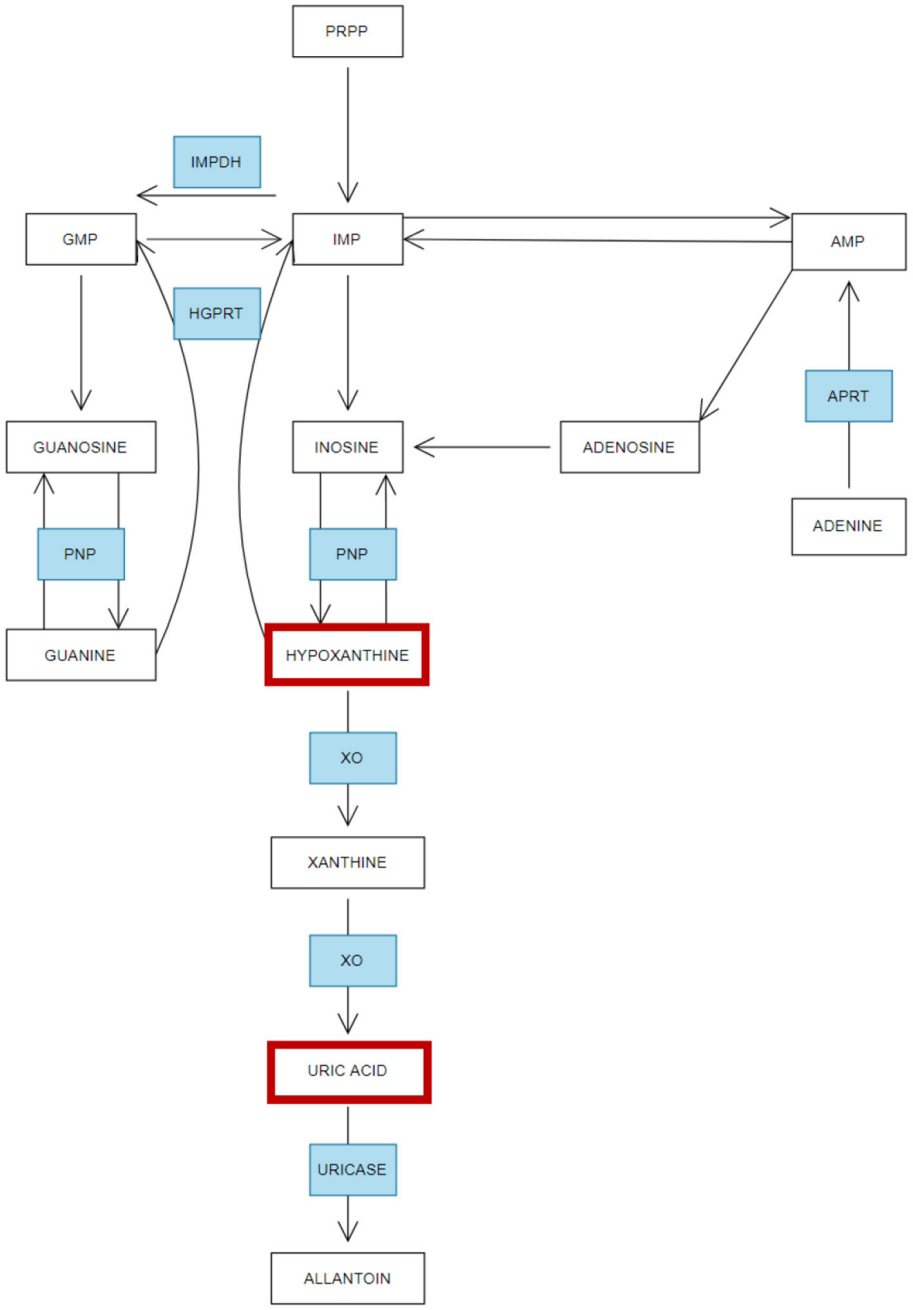
Simplified overview of purine metabolism—Adapted from López-Cruz et al. (2016). Summary of the metabolism of purines and how this process is affected in ME/CFS and Gulf War Syndrome. Enzymes (in blue): inosine monophosphate dehydrogenase (IMPDH), 5-phosphoribosyl 1-pyrophosphate (PRPP), inosine monophosphate (IMP), guanosine monophosphate (GMP), adenosine monophosphate (AMP). Purine nucleoside phosphorylase (PNP), hypoxanthine-guanine phosphorybosil transferase (HGPRT), adenine phosphorybosiltransferase (APRT), xanthine oxidase (XO). Red boxes indicate the metabolites altered in GWS and ME/CFS.

A review of the plasma and serum metabolomics literature indicates consistent dysregulation of multiple pathways in both ME/CFS and GWS, potentially contributing to the shared phenotypic features of these conditions. Notably, many of these pathways are involved in lipid and energy metabolism, which are critical to cellular function and overall metabolic health (Germain et al., 2020; Naviaux et al., 2019; Committee on the Diagnostic Criteria for Myalgic Encephalomyelitis/Chronic Fatigue Syndrome; Board on the Health of Select Populations; Institute of Medicine, 2015; Abdullah et al., 2016; Armstrong et al., 2012; Fluge et al., 2016; Germain et al., 2018, 2022; Hoel et al., 2021; Oberlin et al., 2022; van der Veen et al., 2017; Yamano et al., 2016). For example, Naviaux et al. (2016) conducted a targeted metabolomic analysis to investigate metabolic abnormalities in male participants, including 20 healthy controls (HC), 20 individuals with GWS (diagnosed using both Kansas and CDC criteria), and 22 ME/CFS patients from a previous cohort diagnosed with 2015 Institute of Medicine, CCC, and CDC-1994 diagnostic criteria. A significant finding was the dysregulation of purine metabolism, a pathway that plays dual roles in maintaining intracellular energy homeostasis and mediating extracellular purinergic signaling. These functions are critical for the regulation of chronic pain and inflammation, which are hallmark features of both ME/CFS and GWS (Naviaux et al., 2016, 2019). Downregulation of purine metabolism in serum from both ME/CFS and GWS compared to HC was observed. Purines are key precursors for nucleic acid synthesis, and integral components of essential molecules involved in energy metabolism, such as NADP and NADPH (Figure 4). Additionally, purines function as metabolic signals and play crucial roles in the central nervous system, particularly in the functioning of neuronal and glial cells (Fumagalli et al., 2017). Uric acid, a potent antioxidant and the terminal product of purine metabolism is decreased in serum of GWS (Naviaux et al., 2019). Further evidence of purine metabolism dysregulation comes from a study by McGregor et al. (2019) which analyzed the serum and urine profiles of 46 ME/CFS patients (meeting CCC criteria) and compared them with 26 healthy controls. Their findings indicated that, compared with the healthy controls, there were significant reductions in hypoxanthine serum levels in ME/CFS patients during PEM, a hallmark symptom triggered by physical activity. The reduction in purine levels was associated with increased oxidative stress and endothelial dysfunction, both of which have been implicated in the pathophysiology of both ME/CFS and GWS (Furuhashi, 2020; Ho et al., 2010). In ME/CFS, oxidative stress has emerged as a potential biomarker for fatigue; it results from an imbalance between reactive oxygen species (ROS) production and antioxidant defenses, leading to cellular damage. Elevated levels of oxidative stress markers have been consistently reported in ME/CFS, including malondialdehyde (MDA), isoprostanes, 8-OH-deoxyguanosine, 2,3 diphosphoglyceric acid, thiobutyric acid, and protein carbonyls. These biomarkers provide indirect evidence of oxidative damage to lipids, proteins, and DNA and potentially provide indirect indicators of oxidative stress. In the context of GWS, studies on oxidative stress are limited and its role in GWS remains underexplored (Fukuda et al., 2016). Interestingly, a recent study on patients with prolonged recovery from infectious mononucleosis (caused by the Epstein-Barr Virus EBV) demonstrated dysregulation in nucleotide and glutathione (GSH) plasma levels prior to the onset of the illness, indicating that lower levels of antioxidant capacity may predispose individuals to more severe disease outcomes following viral infection (Jason et al., 2022). Given that both nucleotide and antioxidant pathways are critical in managing oxidative stress, reduced baseline function in these pathways may impair the physiological response to viral infections. This indicates that enhancing antioxidant levels in vulnerable populations—either as a preventative measure before viral exposure or as a long-term therapeutic strategy—could potentially mitigate oxidative stress and improve outcomes for patients with ME/CFS and GWS.

Additional insight into metabolic dysregulation can be gained from the study of lipid pathways. Key lipids, including ceramides, sphingomyelins, phospholipids, diacylglycerols, and triglycerides, as well as branch-chain amino acids, have been found to be dysregulated in both ME/CFS and GWS (Germain et al., 2018, 2020; Naviaux et al., 2019; Armstrong et al., 2012; Fluge et al., 2016; Hoel et al., 2021; Oberlin et al., 2022; Yamano et al., 2016). Sphingomyelins, in particular, are hypothesized to play a critical role in skeletal muscle function, potentially impacting muscle fatigue (Sabbadini et al., 1999); the increase of sphingomyelins pathway products in GWS, and decrease in ME/CFS (Naviaux et al., 2016, 2019), may therefore contribute to the muscle fatigue experienced by patients with these conditions. Energy metabolism and lipid metabolism are intimately connected, with many of the dysregulated lipids identified in these patients being essential components of cellular membranes and having significant roles in energy metabolism (Hu et al., 2022). Mitochondria are responsible for generating Adenosine Triphosphate (ATP) through oxidative phosphorylation, a process that occurs within 5 protein complexes (Complexes I–V) embedded in the inner mitochondrial membrane. These complexes work sequentially to transfer electrons, establish a proton gradient, and drive ATP synthesis. At the core of this process is the electron transport chain (ETC), a series of protein complexes and coenzymes that facilitate the transfer of electrons from energy rich molecules to molecular oxygen. The electron transfer generates the proton gradient necessary for ATP production. The alterations observed in lipid metabolism in patients with GWS and ME/CFS suggest potential mitochondrial dysfunction, which may underlie the profound fatigue characteristic of these conditions (Germain et al., 2018, 2020, 2022; Armstrong et al., 2012; Fluge et al., 2016; Hoel et al., 2021; Oberlin et al., 2022; Yamano et al., 2016). Recent studies have highlighted the role of mitochondrial impairment and altered energy metabolism as key contributors to the pathophysiology of GWS, with their severity correlating to disease progression in 19 veterans suffering from GWS and 17 healthy controls, comparing these factors to markers of peripheral inflammation (Golomb et al., 2023). Mitochondrial function in GWS was investigated using muscle biopsies and three experimental protocols designed to evaluate different aspects of mitochondrial metabolism. These protocols included; (1) Assessing fatty acid oxidation using addition of sequential substrates, (2) Mitochondrial respiration analysis with MiR05 respiration medium and sequential substrates, and (3) Assessment of complex II function using MiR05 supplemented with rotenone and complex II specific substrates. The results revealed a significant reduction in mitochondrial oxidative phosphorylation activity at complexes I and II in GWS patients compared to healthy controls. This impairment was evidence both when glucose was the primary fuel source, and when fatty acids were used alongside the uncoupler carbonyl-p-trifluoromethoxyphenylhydrazone, which bypasses the respiratory control mechanisms. Notably, oxidative phosphorylation efficiency at complexes I and II—specifically when glucose was the fuel source—emerged as a stronger predictor of GWS severity than markers of peripheral inflammation. These findings underscore the critical role of mitochondrial dysfunction in the pathogenesis of GWS and suggest that targeting impaired energy metabolism may offer novel therapeutic avenues.

Studies in GWS subjects have highlighted altered lipid homeostasis as a contributing factor to the abnormal accumulation of lipids, which in turn promotes cellular inflammation. Ceramides, which are elevated in both GWS and ME/CFS, are implicated in the activation of pro-inflammatory cytokines, including interleukin-6 (IL-6) and tumor necrosis factor α (TNF-α) within immune cells such as macrophages, as well as in serum (Germain et al., 2020; Hoel et al., 2021; Oberlin et al., 2022). Moreover, distinct cytokine signatures have been associated with varying severity levels of ME/CFS, where higher levels of plasma pro-inflammatory cytokines, such as colony-stimulating factor 2 (CSF2), correlate with increased physical and fatigue symptoms in these patients (Giloteaux et al., 2023). Elevated ceramides are also known to promote apoptosis (Siskind et al., 2010). The combination of dysregulated lipid homeostasis and impaired purine metabolism may offer a mechanistic explanation for the similar symptoms observed in ME/CFS and GWS. Elevated oxidative stress, alongside metabolic dysregulation and inflammation will disrupt energy metabolism, potentially leading to fatigue and other debilitating symptoms characteristic of these conditions.

Identifying similarities between metabolite alterations in GWS and ME/CFS is an important step toward uncovering potential therapeutic targets and enhancing our understanding of the underlying pathophysiology that contributes to shared symptoms, such as fatigue. Fatigue, while prevalent in both conditions, may be a secondary manifestation driven by distinct underlying metabolic dysfunctions. The observed alterations in energy and lipid metabolism present promising therapeutic avenues for alleviating symptoms in these patient populations. Recent studies have begun to explore these metabolic pathways as potential therapeutic targets. For instance, a study on ME/CFS demonstrated the efficacy of the tricarboxylic acid (TCA) cycle metabolite oxaloacetate in improving symptoms (Cash and Kaufman, 2022). The TCA cycle plays a central role in cellular respiration by generating high energy molecules that fuel oxidative phosphorylation. Similarly, Long COVID, a condition with significant clinical overlaps with ME/CFS (Komaroff and Lipkin, 2023), patients showed marked improvements in fatigue scores as measured by the Chalder Fatigue Questionnaire in a randomized, double-blind, placebo-controlled trial using AXA1125. This compound contains five amino acids—leucine, isoleucine, valine, arginine, and glutamine along with N-acetylcysteine, a precursor in GSH biosynthesis. These components act synergistically to address key mechanisms underlying fatigue and metabolic dysfunction. The amino acids improve mitochondrial bioenergetics by enhancing oxidative phosphorylation and the efficiency of energy production pathways. In addition, N-acetylcysteine replenishes intracellular GSH stores, a critical antioxidant that combats reactive oxygen species and mitigates oxidative stress (Finnigan et al., 2023). Oxaloacetate has also been investigated for therapeutic potential in conditions characterized by chemical liver injury. Its role in scavenging reactive oxygen species (ROS) has been shown to mitigate oxidative damage and preserve mitochondrial integrity (Kuang et al., 2018). Given that both ME/CFS patients and GWS models exhibit elevated levels of oxidative stress, the symptom improvement observed with oxaloacetate treatment may be attributable to its capacity to reduce oxidative stress (Gottschalk et al., 2022; Kodali et al., 2018; Morris et al., 2017; Ribeiro and Deshpande, 2021). AXA1125, which includes the antioxidant N-Acetyl Cysteine, may offer symptom relief through a mechanism similar to that of oxaloacetate, primarily by attenuating oxidative stress and its deleterious effects on cellular function. The safety profile of AXA1125, combined with its potential to improve mitochondrial function and reduce oxidative damage, warrants further investigation in clinical trials in ME/CFS and GWS patients. These trials could provide valuable insights into the effectiveness of targeting oxidative stress and mitochondrial dysfunction as a therapeutic strategy in these conditions.

### ME/CFS and fibromyalgia

Both ME/CFS and FM are characterized by metabolite alterations that suggest dysregulation in energy (Germain et al., 2018, 2020, 2022; Armstrong et al., 2012; Fluge et al., 2016; Hoel et al., 2021; Yamano et al., 2016) and lipid metabolism (Germain et al., 2018, 2020, 2022; Armstrong et al., 2012; Fluge et al., 2016; Hoel et al., 2021; Yamano et al., 2016), FM (da Silva et al., 2021; Menzies et al., 2020) which may contribute to the elevated oxidative stress, inflammation, and metabolic dysfunction observed in these conditions. Here, we focus on key metabolic pathways, with particular emphasis on tryptophan metabolism.

Tryptophan metabolism appears to be differentially altered in ME/CFS and FM, relative to healthy controls, potentially contributing to the symptoms of each condition. Groven et al. (2021) investigated kynurenine metabolites in plasma from 49 ME/CFS patients (meeting CDC-1994 criteria), 57 FM patients, and 54 HC (Groven et al., 2021), while Schwarz et al. (2002) analyzed plasma samples from 17 FM female patients and 17 female HC. Both studies reported differential alternations in tryptophan metabolism. Smaller studies by Georgiades et al. (2003), 11 ME/CFS patients (meeting CVC-1994 criteria) and 11 HC (plasma) and Badawy et al. (2005), 23 ME/CFS (meeting CDC-1994 criteria) and 42 healthy control (serum), similarly evaluated tryptophan levels. In FM, tryptophan levels were consistently reduced compared with healthy controls (Groven et al., 2021; Schwarz et al., 2002; Kavyani et al., 2022) while in ME/CFS tryptophan levels were increased (Groven et al., 2021; Georgiades et al., 2003; Badawy et al., 2005). Despite these differences in tryptophan levels, both conditions are associated with reduced levels of kynurenine, a metabolite of tryptophan (Schwarz et al., 2002; Kavyani et al., 2022). Tryptophan is an essential amino acid involved in the synthesis of melatonin and serotonin; melatonin is critical for regulating the sleep-wake cycle, while serotonin is a neurotransmitter that plays a key role in mood regulation, appetite, pain perception, and sleep. In ME/CFS elevated plasma tryptophan levels may enhance the conversion of tryptophan to 5-hydroxytryptophan in the brain, potentially leading to central fatigue, a core symptom of ME/CFS (Castell et al., 1999). In contrast, the decreased tryptophan levels observed in FM are thought to contribute to lower brain serotonin levels. Serotonin deficiency has been implicated in various aspects of FM symptoms, including sleep disturbances, altered pain perception, and mood disorders. The widespread chronic pain experienced by FM patients may be linked to serotonin's role in muscle function and skeletal muscle fiber growth (Chandran et al., 2012). Tryptophan, a precursor of serotonin, is subject to complex regulation influenced by several factors including the availability of cofactors and competition with other amino acids for transport mechanisms (Anderson et al., 2024; Namkung et al., 2015). Consequently, a higher plasma tryptophan does not necessarily correlate with increased central serotonin synthesis. Moreover, serotonin produced in the gut cannot cross the blood-brain barrier, and therefore, does not contribute directly to central neurotransmission; however, peripheral serotonin can indirectly modulate brain function and influence central neurotransmission through vagal pathways and neuro-endocrine signaling, although this relationship is complex and not fully understood (Sbrini et al., 2022). The permeability of the blood-brain barrier, a key factor in these processes, is influenced by several variables, including the stress response and inflammatory mediators (Tate et al., 2022).

**Figure 5:**
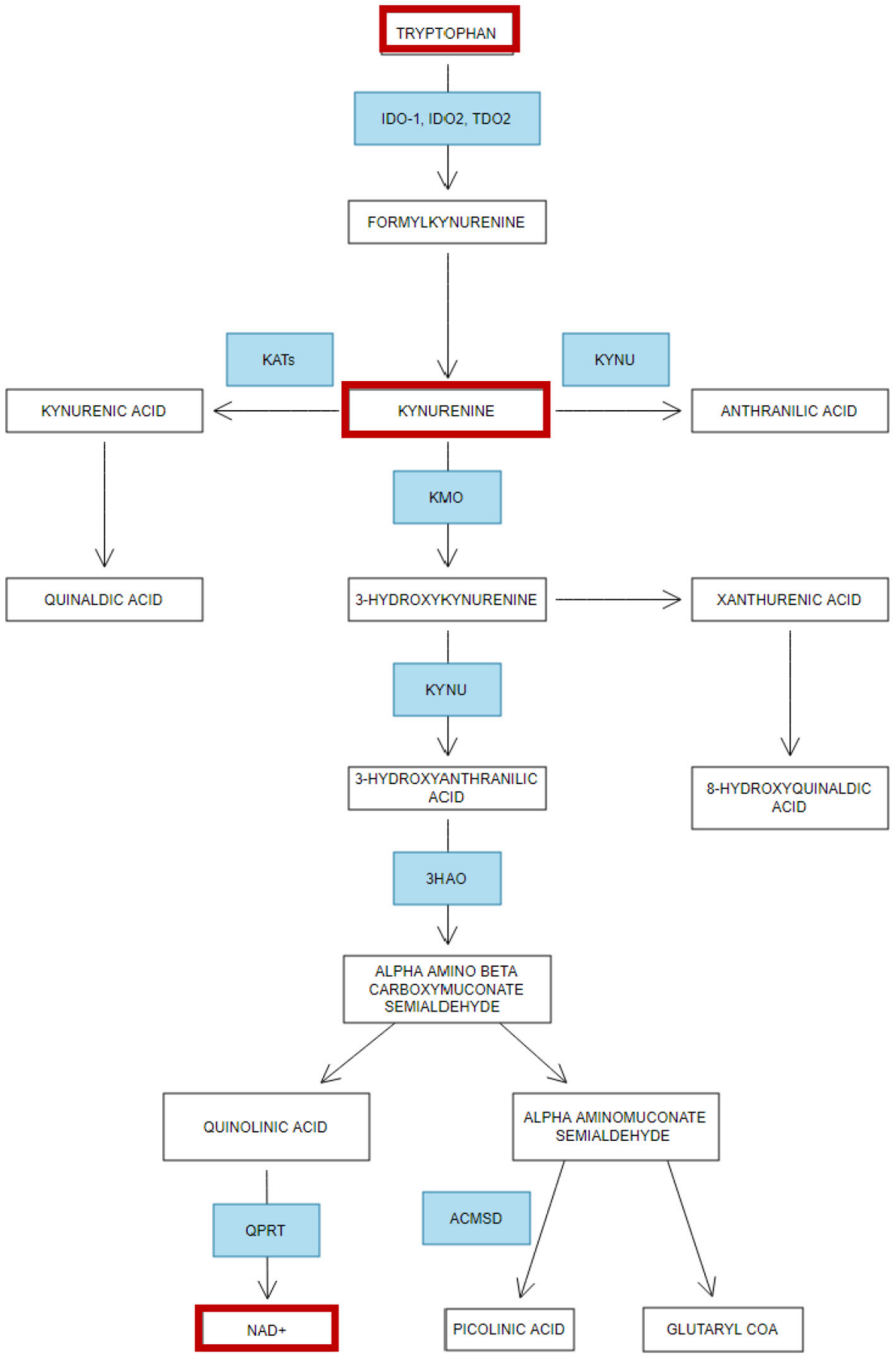
Simplified overview of Tryptophan metabolism—Adapted from Jones et al. (2013). Summary of the metabolism of tryptophan and how it becomes dysregulated in ME/CFS and Fibromyalgia. Enzymes (in blue): 3HAO, 3-hydroxyanthranilic acid oxygenase; IDO, Indoleamine 2,3-dioxygenase; QPRT, quinolinic acid phosphoribosyltransferase; TDO2, Tryptophan 2,3-dioxygenase; KYNU, Kynureninase; KATs, Kynurenine aminotransferases; KMO, Kynurenine 3-monooxygenase; ACMSD, Aminocarboxymuconate-semialdehyde decarboxylase. Red boxes indicate the metabolites altered in fibromyalgia and ME/CFS.

Beyond its role in serotonin synthesis, tryptophan plays a central role in the kynurenine pathway, a metabolic pathway critical for the production of nicotinamide adenine dinucleotide (NAD+), a molecule essential for ATP generation in cellular metabolism (Savitz, 2020) (Figure 5). Activation of the kynurenine pathways is often associated with reduced free circulating (reviewed by Hazrati et al., 2024) and brain tryptophan levels (Hazrati et al., 2024; Clark et al., 2016). The rate-limiting enzyme in this pathway is Indoleamine 2,3-dioxygenase 1 (IDO-1), which is upregulated by pro-inflammatory agents such as interferon-γ (IFN-γ) and tumor necrosis factor-alpha (TNF-α), interleukin-1β (IL-1β), interleukin-12 (IL-12), all cytokines that are released during inflammatory responses (Yang et al., 2024). When IDO-1 is activated, it negatively impacts cellular energy metabolism by catalyzing the conversion of tryptophan into kynurenine, leading to tryptophan depletion. *In vivo*, findings demonstrate that the overexpression of indoleamine 2,3-dioxygenase (IDO) restricts ATP production in CD8+ cells, whilst *in vitro* studies indicate this effect is mediated by reduced activity of electron transport chain complex I, which accounts for the decreased ATP production observed in infiltrating T cells (Liu et al., 2009). As mentioned previously, ATP is the primary energy currency of the cell produced in the mitochondria and is essential for various cellular processes including muscle contraction and other physiological processes.

In addition, ATP serves as a neurotransmitter, co-transmitter, and neuromodulator, undertaking an important role in pain processing, and ATP P1 receptors (A1, A2A, A2B, and A3) are found ubiquitously expressed in the nervous system (Franco et al., 2021). The kynurenine pathway is not only integral to energy production but also serves as a key regulator of the immune system (Savitz, 2020). NAD+ is indispensable for maintaining mitochondrial function (Canto et al., 2015), regulating energy metabolism, and contributing to cellular stress responses and DNA repair processes. A reduction in NAD+ levels has been associated with various metabolic disorders (Sara et al., 2021), underscoring its importance in cellular homeostasis. It also possesses antioxidant properties, potentially through its ability to scavenge free radicals (Kirsch and De Groot, 2001), thereby mitigating oxidative stress. Dysregulation of the kynurenine pathway, as evidenced by low kynurenine levels has been linked to cognitive impairments such as brain fog and memory issues, which are commonly reported in both ME/CFS and FM (Groven et al., 2021; Schwarz et al., 2002; Kavyani et al., 2022). Furthermore, decreased NAD+ levels, potentially a consequence of reduced kynurenine, may contribute to fatigue by impairing energy production, and disrupting the coordination of circadian rhythms necessary for effective sleep (reviewed in Dehhaghi et al., 2022). This potential reduction of NAD+ in ME/CFS and FM could be associated with detrimental effects on cellular function; low NAD+ levels have been implicated in impaired sirtuin activity, a family of proteins that play a critical role in metabolic regulation, epigenetic control of chromatin structure, and mitochondrial function (Katsyuba et al., 2020). Sirtuins are known to influence the body's response to oxidative stress and inflammation, and their dysregulation could lead to increased oxidative damage and reduced ATP production (Sara et al., 2021), further exacerbating symptoms of fatigue and metabolic dysfunction. Moreover, NAD+ is also a key player in redox reactions, and its depletion is thought to contribute to the development of oxidative stress. Conversely, boosting NAD+ levels has been proposed as a potential protective strategy against oxidative damage (Xie et al., 2020). Tryptophan also significantly influences immune function by regulating T cell activity, particularly by modulating cell proliferation. Abnormal levels of tryptophan can therefore negatively impact immune responses, metabolic stress pathways, and brain function, leading to a wide range of symptoms associated with ME/CFS and FM (Gostner et al., 2020). Beyond its immune-related roles, tryptophan is involved in hepatic function, specifically in the synthesis of niacin (vitamin B3), which is essential for energy metabolism and DNA synthesis (Bouma et al., 2016).

Similar to GWS and ME/CFS, disruptions in purine metabolism are suspected to play a role in the pathophysiology of FM. Fais et al. (2013) studied serum from 22 FM patients compared with 22 healthy female controls and identified significant alterations in purine metabolism, specifically decreased levels of adenosine, a precursor for ATP synthesis. Findings from FM align with evidence from ME/CFS research, where model-based studies also suggest reduced adenosine availability. PEM is also thought to be linked to insufficient ATP levels due to a lack of available adenosine, which delays the rapid replenishment of ATP stores post-exercise and may contribute to prolonged recovery times observed in these patients following physical exertion (Lengert and Drossel, 2015). Furthermore, in ME/CFS, PEM has been associated with defects in the TCA cycle, where a metabolic impairment reduces ATP production and increases lactate accumulation (Nilsson et al., 2020). In Fibromyalgia, reduced levels of adenosine are also implicated in pain modulation. Adenosine is known to play a role in pain transmission, and its deficiency may exacerbate pain symptoms in FM patients (Fais et al., 2013). Moreover, adenosine is involved in the regulation of the sleep/wake cycle. Elevated levels of adenosine in certain brain regions, such as the cortex and basal forebrain, have been associated with prolonged wakefulness, while lower levels are observed during sleep recovery periods; this suggests that adenosine may act as a homeostatic regulator of sleep, with higher levels promoting the need for rest (Huang et al., 2014).

Given adenosine's role in energy regulation and neurotransmission, other metabolites involved in cellular metabolism, such as taurine, may also contribute to the disrupted energy balance seen in these conditions. Alterations in taurine levels have been observed in both FM and ME/CFS, with taurine levels being elevated in FM and reduced in those with ME/CFS (Menzies et al., 2020; Germain et al., 2017; Larson et al., 2000; Malatji et al., 2017). Taurine is known to play a significant role in energy metabolism, and dysregulation of taurine levels in these conditions may contribute to the disruption of this process, potentially linking it to the overlapping symptoms observed in both disorders (Wen et al., 2019). Taurine also serves a crucial protective function against oxidative stress, primarily by maintaining GSH stores and reducing inflammation. Its role in supporting mitochondrial function further underscores its importance in cellular energy regulation. Taurine has been shown to mitigate oxidative stress induced by intense physical activity, with studies indicating that taurine supplementation can enhance muscle strength and recovery (Baliou et al., 2021). The dysregulated taurine levels seen in FM and ME/CFS may impair the ability of these patients to effectively manage oxidative stress, exacerbating mitochondrial dysfunction and contributing to the dysregulation of energy metabolism. Furthermore, Germain et al. (2017) identified significantly reduced taurine levels in plasma of ME/CFS patients relative to HC. Taurine plays a pivotal role in muscle function and development, and its depletion in ME/CFS patients may contribute to the metabolic abnormalities observed in this condition. Notably, taurine is essential for conjugating bile acids to form bile salts necessary for proper lipid absorption, and its reduction could contribute to the dysregulation of the bile acid biosynthesis pathway. This dysregulation may have downstream effects on the gut microbiome, which is known to exhibit high levels of dysbiosis in ME/CFS patients (Morten et al., 2018; Nagy-Szakal et al., 2017; Xiong et al., 2023). The common alterations in key metabolic pathways in both FM and ME/CFS suggest a reduced capacity to manage oxidative stress which may underlie the energy metabolism dysfunction and similar symptoms experienced by patients with these conditions. A pivotal study by Germain et al. (2022) explored the impact of vigorous exercise on ME/CFS patients compared with healthy controls, using two intense exercise assessments conducted 24 h apart. The study's methodology was designed to induce PEM in ME/CFS patients, allowing for an examination of the metabolic differences in plasma between patients and controls, particularly in energy metabolism. The most significant disparities emerged during the recovery phase after the first exercise challenge, where pathway analysis revealed significant changes in numerous metabolic pathways, including the urea cycle, and glutamate and pyruvate metabolism. These results suggest a metabolic shift in ME/CFS patients from a hypometabolic state at baseline, characterized by down-regulated pathways to a hypermetabolic state post-exercise, characterized by over-activated pathways. A similar metabolic response was observed by McGregor et al. (2019) in serum following a PEM challenge. This study also identified the depletion of the purine metabolite, hypoxanthine, in chronic fatigue patients (according to Canadian Consensus criteria) which may impair ATP production and contribute to the biochemical changes associated with PEM. Together, these findings highlight a distinct metabolic signature in ME/CFS, where energy production pathways may fail to respond efficiently to physical exertion, potentially driving exacerbation of symptoms seen in PEM.

## Analysis using proteomics

Proteomics, the comprehensive analysis of the entire protein complement within a biological sample, such as blood cells or plasma, offers valuable insight into disease-specific biomarkers (Coorssen, 2022). By establishing baseline proteomic profiles using healthy controls, researchers can identify biomarkers that are specific to conditions like ME/CFS, Fibromyalgia, and GWS. Peripheral blood mononuclear cells (PBMCs) are a diverse group of immune cells including lymphocytes (T cells, B cells, and NK cells) and monocytes, that play a role in immune function and inflammation. Recent studies have focused on the proteomes of peripheral blood mononuclear cells (PBMCs) from ME/CFS patients, using Sequential Window Acquisition of All Theoretical Mass Spectra (SWATH-MS) for detailed protein analysis (Sweetman et al., 2020). This approach identified 60 proteins with differential expression between ME/CFS patients and matched healthy controls, effectively distinguishing the patient group from the controls. Among the proteins with increased expression in ME/CFS were 3-hydroxybutyrate dehydrogenase type 1 (BDH1) and acetyl-CoA acetyltransferase 2 (ACAT2), both of which are integral to fatty acid metabolism and processing of ketone bodies. Another protein of interest, SLC25A11, was also found to be upregulated in the PBMCs of ME/CFS patients compared with controls. SLC25A11 belongs to the solute carrier family 25 (SLC25), which encompasses 53 known carrier proteins that play critical roles in regulating cellular metabolism and physiological processes (reviewed in Kunji et al., 2020). Specifically, SLC25A11 functions as the mitochondrial oxoglutarate carrier, facilitating the exchange of cytosolic malate for 2-oxoglutarate from the mitochondrial matrix. This carrier protein is pivotal in several metabolic pathways, including the malate-aspartate shuttle, the oxoglutarate-isocitrate shuttle, and gluconeogenesis (Iacobazzi et al., 1992). The upregulation of SLC25A11 could have several implications for cellular metabolism in ME/CFS. Increased mitochondrial import of malate may enhance complex I activity, leading to elevated mitochondrial ATP production. Conversely, the increased export of 2-Oxoglutarate into the cytosol could augment the activity of Fe (II) 2-oxoglutarate (2OG)—dependent oxygenases, enzymes that catalyze a variety of critical reactions including hydroxylation, halogenation, desaturation, and ring transformation. These enzymes are involved in nutrient sensing and the cellular response to stress, including hypoxia, and are implicated in various disease processes (reviewed Fletcher and Coleman, 2020). The observed increase in mitochondrial malate and cytosolic 2-oxoglutarate through elevated SLC25A11 expression aligns with the systemic energetic dysregulation and oxidative stress reported in plasma metabolomic studies in ME/CFS patients.

In ME/CFS, in addition to PBMCs, proteomics analyses show that plasma proteins associated with the extracellular matrix, immune system, and cell-cell communication differ significantly from healthy controls. Notably, the ephrin pathway, which regulates cell signaling, cellular morphology, stem cell niche maintenance, axon guidance, and energy metabolism was found to be upregulated in ME/CFS patients. The ephrin pathway plays a crucial role in immune function and inflammatory responses and proteomics studies further support evidence of chronic inflammation, perhaps chronic infection (Milivojevic et al., 2020) and immune dysregulation (Giloteaux et al., 2023), with additional links to coagulation abnormalities and chronic infection (Nunes et al., 2022). Key studies in ME/CFS include Germain et al. (2021) who analyzed plasma proteomes from 20 female ME/CFS patients (CDC-1994 criteria) and 20 healthy controls, finding all significantly altered proteins to be elevated in ME/CFS. A larger study by the Hanson group employed machine learning to distinguish ME/CFS patients from healthy controls with 79.1% accuracy (AUROC value 0.891) using only seven proteins (Giloteaux et al., 2023). These proteins included extracellular vesicle interleukin-2 (EV IL2) and cathelicidin antimicrobial protein (CAMP) which were elevated, while immunoglobulin lambda variable 1-47 (IGLV1-47), cartilage acidic protein 1 (CRTAC1), leucine-rich alpha-2-glycoprotein 1 (LRG1), insulin-like growth factor 1 (IGF1), and tubulin alpha-1 (TUBA1) were decreased relative to healthy controls. In a study by Milivojevic et al. (2020) 39 ME/CFS (CDC-1994 and/or CCC criteria) were compared to 41 healthy controls, 12 patients had a very high level, while 3 had very low levels of IGHV3-23/30 protein.

In FM, a systematic review by Gkouvi et al. (2024), explored ten studies comparing proteomic differences in saliva, plasma, serum, and cerebrospinal fluid (CSF) relative to healthy controls. Differences were observed in levels of transferrin, α-, β-, and γ-fibrinogen chains, profilin-1, transaldolase, PGAM1, apolipoprotein-C3, complement C4A and C1QC, immunoglobin fragments, and acute-phase reactants. In saliva, studies by Ciregia et al. (2019) 30 FM, 30 HC and Bazzichi et al. (2009) 22 FM and 26 healthy controls, elevated levels of transaldolase (TALDO) were observed in FM patients, saliva and serum. Upregulation of TALDO, an enzyme linked to the pentose phosphate pathway enzyme which generates NADPH a key co-factor involved in anti-oxidant pathways, could suggest heightened levels oxidative stress (Ciregia et al., 2019; Bazzichi et al., 2009). Additionally, calgranulin A, a calcium-binding S100 protein that binds calcium and belongs to the S100 protein family that regulates various intracellular processes was also increased relative to healthy controls in a serum study by Hsu et al. (2022), 30 FM and 25 healthy controls. S100 plays a role in dealing with oxidative stress hence this increase is perhaps indicative of increased oxidative stress (Hu and Lin, 2021). Glucose levels were also raised in FM patients relative to healthy controls which might be linked to abnormal energy metabolism. In plasma and serum, markers of coagulation (increased), complement (decreased), and fibrinolysis (increased and decreased) showed changes relative to HC (Gkouvi et al., 2024). In FM markers of elevated inflammation and immune dysregulation were observed on multiple platforms relative to HC which is very similar to the situation observed in ME/CFS (see earlier sections). The similarity between overlap in the pathophysiology of ME/CFS and FM was demonstrated by Schutzer et al. (2023), who analyzed CSF proteomes from 15 ME/CFS patients and 15 ME/CFS patients with FM comorbidity. In ME/CFS (*n* = 15) and FM (*n* = 15) samples no significant differences between the two groups was observed although both were very different from the healthy control group (Schutzer et al., 2023). Schutzer et al. (2023) did not show any significant differences in the CSF proteome by adding in the FM diagnosis. Overlap in the underlying biology was also observed in CFS and GWS by Baraniuk et al. (2005) and Johnson et al. (2016). Studying two cohorts of CFS (*n* = 10) and GWS patients (*n* = 10), one pooled and the other looking at individual cases, Baraniuk found very similar CSF proteome differences relative to a healthy control group (*n* = 10) (Baraniuk et al., 2005). With chronic fatigue being a key element of GWS, a distinct proteome was observed that was common to both ME/CFS and GWS patient groups relative to healthy controls. Certain proteins were present at much higher levels in the CFS groups with the authors suggesting that decreased plasma efflux of brain-related proteins could lead to the build-up in CFS/GWS. This may be linked to blood-brain barrier dysfunction or changes to sleep patterns in these patient groups. In mice exposed to Gulf War agents, brain tissue showed evidence of mitochondria disturbances, with astroglia and microglia activation in the hippocampus with significant reductions in Cardiolipin (Abdullah et al., 2016), a strong indicator of decreased activity of the electron transport chain (Shen et al., 2015). Evidence of lipid dysregulation and energy issues was also observed in plasma, with elevated odd-chain acylcarnitines in GW agent-exposed mice, and in veterans with GWI, relative to their respective controls. Compared with the aforementioned plasma differences, here there were no differences in the brain concentrations of odd-chain acylcarnitines between control and exposed mice. As such, plasma changes in these acylcarnitines suggest that these effects are likely to be peripheral rather than central manifestations of this illness (Abdullah et al., 2016).

In mouse models simulating GWS, mice were exposed to pyridostigmine bromide (PB) and permethrin (PER) for 10 days at 12 weeks of age, with proteomic analysis of brain extracts conducted at 5 months post-exposure. The results indicated both mitochondrial dysfunction and dysregulation of immune and inflammatory responses. Specifically, brain tissue analysis revealed decreased levels of respiratory chain complex I, II, IV, and V suggesting damage to mitochondrial proteins. This damage likely accelerates protein turnover, thereby impairing the energy-generating capacity of mitochondria. This aligns with previous plasma metabolomics profiles observed in GWS, where dysregulation of energy metabolism was evident. The failure of these mitochondrial complexes in brain tissue is also expected to increase ROS production leading to further localized cellular damage. Although an inflammatory phenotype was not detected in the brain, systemic immune dysregulation was suggested by significant decreases of IFNγ, TNFα, IL-1β, and IL10 (Zakirova et al., 2017). Proteomic studies in FM have also highlighted dysregulation in stress response, muscle contraction, and immune pathways. Ghafouri et al. (2023) used microdialysis to identify 26 interstitial muscle proteins associated with these pathways, with most proteins being downregulated. Plasma protein expression in FM has been correlated with pain severity, with an upregulation of proteins involved in immune and inflammatory processes, including upregulation of ceruloplasmin, a key acute-phase reactant (Ghafouri et al., 2023). Maldonado-García et al. (2021) employed tandem mass tag proteomics (TMT) and mass spectrometry (MS) to analyze PBMC, identifying differential expression of proteins regulated by the liver X receptor-retinoid X receptor (LXR/RXR) pathway, which modulates lipid metabolism and inflammation, potentially influencing the expression of pro-inflammatory cytokine genes (Schulman, 2017). Hence, proteomics can be a powerful tool for elucidating the molecular mechanisms underlying FM and identifying potential therapeutic targets.

### MRI and MRSI techniques

Magnetic resonance imaging (MRI) and magnetic resonance spectroscopy imaging (MRSI) are powerful non-invasive techniques that can be used to study tissue biomarkers and complement plasma metabolomics studies. These imaging methods have provided insights into the neurobiological alterations associated with ME/CFS, GWS, and FM. In a study by Baraniuk (2022), MRI scans were conducted on the brains of ME/CFS patients, GWS patients, and healthy controls both at rest and while doing cognitive tasks. At rest, no significant differences were observed between the GWS and control groups. However, the ME/CFS patients showed a slightly lower blood oxygenation level-dependent (BOLD) signal compared with controls, establishing a baseline difference. Post-exercise, ME/CFS patients showed a significant increase in BOLD across various brain regions, suggesting a global change in brain metabolism in response to the exercise challenge. In contrast, GWS patients experienced a decrease in BOLD in specific regions including the dorsal midbrain, right middle insula, and left Rolandic operculum. This could indicate that, while both conditions involve neurobiological changes, the patterns of alteration differ possibly reflecting distinct underlying mechanisms. The more generalized response in ME/CFS may be linked to systemic metabolic dysregulation following viral infections, whereas the localized changes in GWS could be associated with the neurotoxic effects of medications taken or exposure to toxic substances during the Gulf War (Baraniuk, 2022).

Magnetic resonance spectroscopy imaging (MRSI) studies have provided further evidence of metabolic dysfunction in ME/CFS, particularly in the form of increased ventricular lactate levels. Elevated lactate implies potential mitochondrial dysfunction, with an increased reliance on glycolysis for ATP production (Mathew et al., 2009; Murrough et al., 2010; Shungu et al., 2012). This shift in energy metabolism is consistent with mitochondrial impairment, which forces cells to rely more heavily on glycolysis, producing lactate as a by-product. Shungu et al. (2012) explored the relationship between cortical GSH levels, ME/CFS symptoms, and oxidative stress. The study found significantly lower cortical GSH levels in ME/CFS patients compared with healthy controls. Given that GSH is essential for cellular redox reactions and the processing of ROS, evidence points to an impaired antioxidant defense in ME/CFS (Rae and Williams, 2017). Cortical GSH, which can be measured using MRSI, serves as a marker of the brain's antioxidant capacity. Impaired GSH function is associated not only with neurodegenerative diseases, like Alzheimer's disease, but also with broader issues related to brain metabolism and oxidative stress (Iskusnykh et al., 2022). The elevated lactate levels observed in the brains of ME/CFS patients have important implications for understanding the pathophysiology (Natelson et al., 2017). Lactate and hydrogen ions (H+) are produced during glycolysis and are associated with mitochondrial failure and hypoxia (Ivashkiv, 2020). As lactate and H+ accumulate, blood pH decreases, leading to increased acidity, which can exacerbate oxidative stress and ROS production (Li et al., 2022). Moreover, lactate is a critical energy substrate for astrocytes and neurons, and its dysregulated metabolism in ME/CFS and FM patients may reflect an impaired ability to use lactate effectively for energy (Bélanger et al., 2011). This impaired lactate metabolism could also disrupt the positive adaptations typically associated with regular physical activity, such as mitochondrial biogenesis (Li et al., 2022; Brooks et al., 2022) further contributing to the fatigue and PEM experienced by these patients. Ghali et al. (2019) suggested that resting blood lactate levels could serve as a marker for the severity of ME/CFS. Their study found that 44.7% of ME/CFS patients had elevated blood lactate at rest which was correlated with increased severity of PEM, although this study did not see a correlation with fatigue severity (Ghali et al., 2019). Additionally, ventricular CSF lactate levels, measured by MRSI have been used to compare FM and ME/CFS (Natelson et al., 2017). Mean CSF lactate levels were higher in ME/CFS and FM patients compared with HC, but these measures did not sufficiently distinguish between the two conditions. This discovery suggests that similar underlying pathological processes may be present in both ME/CFS and FM.

## Discussion

ME/CFS, GWS, and FM are three chronic conditions presenting with overlapping symptoms, which lack definitive diagnostic tests and effective pharmacological treatments. Despite their prevalence, the pathophysiological mechanisms underlying these conditions remain poorly understood. One of the key proposed contributors to these conditions is oxidative stress, which is thought to impair energy production in muscles, leading to the characteristic fatigue observed in these patients. For instance, muscle biopsies from ME/CFS patients have revealed cellular membrane damage, likely attributable to elevated levels of ROS (Pietrangelo et al., 2018). Emerging techniques such as metabolic profiling, proteomics, and MRI have the potential to elucidate the factors contributing to elevated ROS levels, offering new avenues for therapeutic intervention, not only in ME/CFS but also in GWS and Fibromyalgia.

A critical question in understanding these conditions is how the observed metabolite differences in ME/CFS, Fibromyalgia, and GWS compare with those seen in other diseases associated with chronic fatigue or cognitive decline. Alzheimer's disease (AD), a condition characterized by well-established cognitive impairment, provides a useful comparison. The Nakayama Alzheimer's disease study demonstrated that cognitive function in older adults, including controls, patients with mild cognitive impairment, and those with AD, was linked to alterations in plasma metabolites such as reduced levels of lysine, ornithine, and uracil (Ozaki et al., 2022). Furthermore, energy metabolism impairments, particularly in the tricarboxylic acid (TCA) cycle, were also implicated in AD (Sun C. et al., 2020; Sun Y. et al., 2020). Similarly, in bipolar disorder, particularly during depressive episodes, patients exhibit altered energy metabolism marked by increased lactate levels and decreased glucose and glycine levels, which parallels results in ME/CFS that suggest TCA impairment and energy metabolism dysregulation (Yamano et al., 2016; Armstrong et al., 2015; Che et al., 2022; Nagy-Szakal et al., 2018).

Reduced physical activity is a common feature of ME/CFS, FM, and GWS. Physical exercise is known for its widespread effects on human physiology, and its impact has been extensively studied, both acutely and over the long term (reviewed in Kelly et al., 2020). Interestingly, the plasma amino acid profiles of athletes after exercise were characterized by decreased levels of leucine, isoleucine, asparagine, methionine, lysine, glutamine, alanine, and certain lipids, which resemble the baseline profiles of ME/CFS patients (Nieman et al., 2013). This similarity suggests that the altered energy metabolism at baseline observed in ME/CFS may compromise the ability of these patients to respond to exercise in the same way as healthy individuals. The depletion of amino acids at baseline in ME/CFS, coupled with their impaired glucose metabolism, could limit the availability of alternative energy sources during exertion, potentially contributing to PEM. The hypermetabolic state observed post-exertion in ME/CFS patients, as described by Germain et al. (2022) and McGregor et al. (2019), underscores the metabolic basis of PEM (Germain et al., 2022; McGregor et al., 2019; Glass et al., 2023). PEM is a debilitating aspect of all three conditions, leading patients to pace their activities to avoid triggering these symptoms (Stussman et al., 2023; Vernon et al., 2023). The distinct physical activity patterns observed in ME/CFS, characterized by reduced activity intensity but not increased sedentary behavior, further highlight the link between PEN and metabolic dysfunction, likely driven by issues with energy metabolism (Newton et al., 2011).

Oxidative stress, characterized by an imbalance between ROS generation and antioxidant defenses, has been implicated in the pathology of ME/CFS, FM, and GWS (Gottschalk et al., 2022; Kodali et al., 2018; Morris et al., 2017; Ribeiro and Deshpande, 2021; Cordero et al., 2011; Dos Santos et al., 2022). Elevated ROS levels can lead to mitochondrial DNA damage, enzyme inhibition, and cellular dysfunction, ultimately contributing to disease progression (Sharifi-Rad et al., 2020). The metabolite profiles of ME/CFS, FM, and GWS suggest that many of the altered metabolites are involved in oxidative stress, either exacerbating ROS production or depleting key antioxidant compounds needed to mitigate oxidative damage. Mitochondria, as the primary source of ROS in mammalian cells, are particularly vulnerable, with oxidative stress contributing to mitochondrial dysfunction, energy depletion, and cell death (Cui et al., 2012).

Several studies have highlighted oxidative stress as a key factor in ME/CFS. For example, Jammes et al. (2012) found elevated plasma markers of oxidative stress in ME/CFS patients both at rest and following exercise (Wood et al., 2021). Castro-Marrero et al. (2013) further demonstrated elevated oxidative damage and reduced antioxidant levels, particularly Coenzyme Q10, in ME/CFS patients. This systemic oxidative stress is thought to contribute to mitochondrial dysfunction, activation of inflammatory pathways, and dysregulation of brain function, potentially explaining symptoms, such as fatigue and cognitive dysfunction (Wood et al., 2021). In fibromyalgia, oxidative stress has also been linked to pathophysiology (Cordero et al., 2010) with studies suggesting that antioxidant therapies may help reduce elevated pro-oxidative pathways such as lipid peroxidation and mitophagy (Assavarittirong et al., 2022). Similarly, GWS has been associated with elevated oxidative stress (Shetty et al., 2017) with melatonin being shown to improve brain function in the GWS model, through modulation of oxidative stress (Madhu et al., 2021).

The importance of oxidative stress in chronic disease is further exemplified by Long COVID, a condition that has emerged following the COVID-19 pandemic and shares many clinical features with ME/CFS (Astin et al., 2023). Oxidative stress induced by viral infection is thought to disrupt the normal redox state of host cells, leading to reduced GSH stores and increased ROS production (De Flora et al., 2020). Similar reductions in GSH have been observed in ME/CFS patients, further supporting the link between oxidative stress and chronic fatigue conditions (Jason et al., 2022; Jarrott et al., 2022). ROS-induced mitochondrial dysfunction and DNA damage, along with the inhibition of key redox transcription factors such as Nuclear Factor Erythroid 2-Related Factor 2 (NRF2) may contribute to the persistence of symptoms in both ME/CFS and Long COVID (Jarrott et al., 2022). ME/CFS has been associated with various viral infections, and the efficiency of antioxidant defense systems may determine whether individuals develop chronic fatigue syndromes following infection (Rasa et al., 2018).

The presence of fibrinolysis-resistant micro-clots in the vasculature of Long COVID patients, as described by Pretorius et al. (2021) provides another potential link between oxidative stress and chronic illness. These micro-clots, which trap inflammatory molecules and prevent proper oxygen exchange in microcapillaries, may contribute to fatigue and other symptoms seen in Long COVID (Baker et al., 2023; Kruger et al., 2022). Similar findings in ME/CFS patients suggest that micro-clots and platelet hyperactivation could also play a role in the pathology of this condition, potentially driven by elevated oxidative stress (Nunes et al., 2022). The hypoxia caused by these micro-clots could exacerbate ROS generation and oxidative stress, creating a vicious cycle that perpetuates the disease state (El Haouari, 2019; Kell and Pretorius, 2022).

Oxidative stress, driven in part by metabolite and proteome alterations, appears to play a central role in the pathology of ME/CFS, GWS, and FM. Mitochondrial dysfunction reduced NAD+ production, and inflammation, including neuroinflammation, are likely contributors to the phenotypes of these conditions (Tate et al., 2022; Morris and Maes, 2014). Dysregulated lipid metabolism, altered lactate levels, and impaired energy metabolism are key factors that may exacerbate ROS levels. This review highlights the potential of metabolomics, proteomics, miRNA, and MRI studies to advance our understanding of chronic diseases, and identifies key areas, such as the impact of altered lipid and energy metabolism, that warrant further investigation. Addressing these metabolic disruptions could lead to the development of more effective therapeutic strategies for managing ME/CFS, GWS, and FM.

## Conclusion

The SARS-CoV-2 pandemic has contributed to a dramatic rise in cases of ME/CFS, underscoring the pressing need for robust and sustained research into chronic conditions such as ME/CFS, FM, and GWS. As the long-term consequences of this pandemic continue to unfold, an investment in both clinical and basic research infrastructure is critical to address the growing burden of these illnesses that profoundly impact millions of individuals worldwide. This review highlights significant overlap in the molecular and cellular disruptions observed across ME/CFS, FM, and GWS. Common pathways implicated in these conditions include dysregulated lipid metabolism, oxidative stress, and impaired energy metabolism, reflecting widespread dysfunction in fundamental biological processes; however, it remains unclear whether these alterations are primary divers of disease or secondary effects resulting from chronic illness. Interventions targeting energy metabolism, while yielding modest benefits, suggest that these abnormalities are likely downstream consequences of other underlying mechanisms. The immune system appears to play a pivotal role in these conditions, with evidence of T-cell and B-cell dysfunction, including signs of exhaustion in ME/CFS. A critical unanswered question is whether this immune dysregulation is a carryover from an initial infection, or whether it reflects ongoing chronic immune activation infection or inflammation when the initial infection has resolved. Investigating the mechanisms underlying PEM, a hallmark feature of ME/CFS, may provide valuable insight into the drivers of symptom exacerbation and the broader pathophysiology of these conditions. Emerging studies using CSF and advanced neuroimaging techniques, such as brain magnetic resonance spectroscopy (MRS) are beginning to uncover neurobiological changes that may further elucidate disease mechanisms. Notably, the overlap in CFS abnormalities observed across chronic conditions suggests a potential shared pathway of brain dysfunction. Moving beyond broad plasma-based analyses to focus on specific organ systems, such as the brain, liver, and kidney, is likely to yield more targeted insights. Improved diagnostic tools are essential to accurately strategy patient subgroups and reduce the risk of misclassification, which can obscure the interpretation of study findings. Addressing these complex challenges requires coordinated international efforts and substantial financial support. By committing to the development of a robust research framework, it may be possible to unravel the causal pathways driving these illnesses, distinguish subgroups of patients with shared mechanisms, and deliver targeted interventions. Failure to act risks exacerbating the significant health, social, and economic burden posed by these conditions. Conversely, sustained and focused research efforts have the potential to transform our understanding of these disorders and significantly improve patient outcomes on a global scale.

## Glossary

2OG, 2-oxoglutarate; ACAT2, Acetyl-CoA acetyltransferase 2; AD, Alzheimer's disease; AMP, Adenosine monophosphate; APRT, Adenine phosphorybosiltransferase; BDH1, 3-hydroxybutyrate dehydrogenase type 1; BOLD, Blood oxygenation level-dependent; CAMP, Cathelicidin antimicrobial protein; CCC, Canadian consensus criteria; CMI, Chronic multisymptomatic Illness; COPIND, Chronic organophosphate induced neuropsychiatric disorder; CRTAC1, Cartilage acidic protein 1; CSF, Cerebrospinal fluid; CSF2, Colony-stimulating factor 2; ETC, Electron transport chain; EV IL2, Extracellular vesicle interleukin-2; FM, Fibromyalgia; GC-MS, Gas chromatography-mass spectrometry; GMP, Guanosine monophosphate; GSH, Glutathione; GWS, Gulf war syndrome; H+, Hydrogen ions; HGPRT, Hypoxanthine-guanine phosphoribosyl transferase; HMDB Ver 5.0, Human metabolome database version 5.0; HPLC, High-performance liquid chromatography; ICD-11, International classification of diseases most recent iteration; IDO, Indoleamine 2,3-dioxygenase; IDO-1, Indoleamine 2,3-dioxygenase 1; IFN-γ, Interferon-γ; IGF1, Insulin-like growth factor 1; IGLV1-47, Immunoglobulin lambda variable 1-47; IL-12, Inteleukin-12; IL-1β, Interleukin-1β; IL-6, Interleukin-6; IMP, Inosine monophosphate; IMPDH, Inosine monophosphate dehydrogenase; LC, GC, Liquid or gas chromatography; LRG1, Leucine-rich alpha-2-glycoprotein 1; LXR/RXR, Liver X receptor-retinoid X receptor; MDA, Malondialdehyde; ME/CFS, Myalgic encephalomyelitis/chronic fatigue syndrome; MiR05, Mitochondrial respiration medium; miRNAs, MicroRNAs; MRI, Magnetic resonance imaging; MRSI, Magnetic resonance spectroscopy imaging; MS, Mass spectrometry; NAD+, Nicotinamide adenine dinucleotide; NMR, Nuclear magnetic resonance; NRF2, Nuclear factor erythroid 2-related factor 2; PB, Pyridostigmine bromide; PBMCs, Peripheral blood mononuclear cells; PEM, Post-exertional malaise; PER, Permethrin; PNP, Purine nucleoside phosphorylase; POTS, Postural orthostatic tachycardia syndrome; PRPP, 5-phosphoribosyl 1-pyrophosphate; ROS, Reactive oxygen species; SLC25, Solute carrier family 25; SWATH-MS, Sequential window acquisition of all theoretical mass spectra; TALDO, Transaldolase; TCA, Tricarboxylic acid cycle; TMT, Tandem mass tag; TNF-α, Tumour necrosis factor αlpha; TUBA1, Tubulin alpha-1; XO, Xanthine oxidase.
